# A telehealth integrated asthma-COPD service for primary care: a proposal for a pilot feasibility study in Crete, Greece

**DOI:** 10.1186/1756-0500-3-198

**Published:** 2010-07-15

**Authors:** Ioanna G Tsiligianni, Thys van der Molen, Nikolaos E Tzanakis, Nikolaos M Siafakas, Ellen van Heijst, Christos Lionis

**Affiliations:** 1 Agia Barbara Health Care Center, Heraklion, Crete, P.O 70003, Greece; 2 Department of General Practice University Medical Centre Groningen, Antonius Deusinglaan 1, P.O 9700 AD, Groningen, The Netherlands; 3 Clinic of Social and Family Medicine, Medical School, University of Crete, Heraklion, Crete, P.O 71003, Greece; 4 Department of Thoracic Medicine, Medical School, University of Crete, Heraklion, Crete, P.O 71003, Greece; 5 Northern Laboratory service for General practitioners Labnoord, Damsterdiep 191, P.O 9713 EC, Groningen, The Netherlands

## Abstract

**Background:**

Chronic obstructive pulmonary disease (COPD) and asthma are considered underdiagnosed and misdiagnosed chronic diseases. In The Netherlands, a COPD-asthma telemedicine service has been developed to increase GPs' ability to diagnose and manage COPD and asthma. A telemedicine COPD-asthma service may benefit Greece as it is a country, partly due to its geography, that does not have easy access to pulmonologists.

**Findings:**

Therefore, a pilot feasibility study has been designed in Greece in order to establish this telemedicine service. Ten rural practices, in the island of Crete, with an average population of 2000 patients per practice will pilot the project supported by three pulmonologists. This paper presents the translated interfaces, the flowcharts and the steps that are considered as necessary for this feasibility study in Crete, Greece.

## Background

Chronic obstructive pulmonary disease (COPD) and asthma are common diseases with significant prevalence in the general population [1,2]. COPD and asthma consultations represent a large proportion of the total primary care consultations [3,4]. Studies have reported a considerable number of underdiagnosed and misdiagnosed cases in primary care clinical settings worldwide [1,5].

Despite the high prevalence of COPD and asthma in Greece [6,7], these diseases are thought to be underdiagnosed [6]. Greece is a country with more than 200 inhabited islands, with the smallest islands having high percentages of elderly residents. Most of the islands lack regular secondary care access. In the mainland, there are a lot of rural and remote areas that also lack easy access to secondary care assistance. Often the General Practitioner (GP) or, at times, a non certified physician are the only health care providers for these patients. In addition, integration of primary health care in the country is not currently a high priority in the health care agenda [8].

Good quality spirometry is the key to the management of these common respiratory conditions and can be accomplished in primary care [9,10]. However, the lack of availability of spirometry and lack of knowledge and training regarding interpretation of spirometric results have been cited as barriers to the early diagnosis and treatment of COPD and asthma in primary care [11]. An additional barrier identified is the lack of secondary care support and availability of specialized physicians (pulmonologists) to general practitioners (GPs), especially in remote and rural areas. In that field telemedicine services have been proved a great help [12,13].

Telemedicine involves the transfer of medical information via telecommunication technologies for the purpose of consulting or to aid in remote medical procedures or examinations http://en.wiktionary.org/wiki/telemedicine. In remote and rural areas, telemedicine services have been shown to be effective in supporting healthcare [12,13]. Services have been successfully implemented to help GPs in the diagnosis and management of several diseases such as asthma, diabetes, skin disorders and emergency cases [14-17].

In The Netherlands, a telemedicine supported asthma-COPD service was developed for increasing the diagnosis and improving the management of asthma and COPD patients in primary care. The service assessed 1022 patients through a telemedicine collaboration of GPs with pulmonologists from March 2007 to November 2008. It identified 182 patients with COPD (18%), 557 patients with asthma (55%) and 103 patients with combined asthma and COPD (10%). In 180 patients (10%), the diagnosis was unclear or lung function tests were not possible or other diagnoses (except of asthma and COPD) were made. Level of disease control was also examined with 47% of asthma patients and 28% of combined asthma-COPD patients identified as well-controlled. Twenty seven percent of the COPD patients were unstable (unpublished data). This service has improved diagnosis and management of COPD and asthmatic patients in The Netherlands.

Although telemedicine services related to primary health care have been tested in Greek islands there has been nothing specifically for COPD or asthma [18]. We would argue that development of a COPD-asthma telemedicine service in Greece could be an effective provision for GPs and patients. Telemedicine offers the possibility of reducing consultations with secondary care and delays in communication and treatment [14-17], issues that are extremely important for Greece due to its particular geographical situation.

This study aims to carry out a feasibility study in Crete, Greece using the framework of the telemedicine service previously developed and established in The Netherlands. If the service works as well as in The Netherlands, attempts to expand the service nationally will be made, subject to government support.

The primary objective of this study will be to evaluate the feasibility and acceptability of the Dutch asthma-COPD telemedicine service in Crete.

## Design of the feasibility study

**Figure 1:**
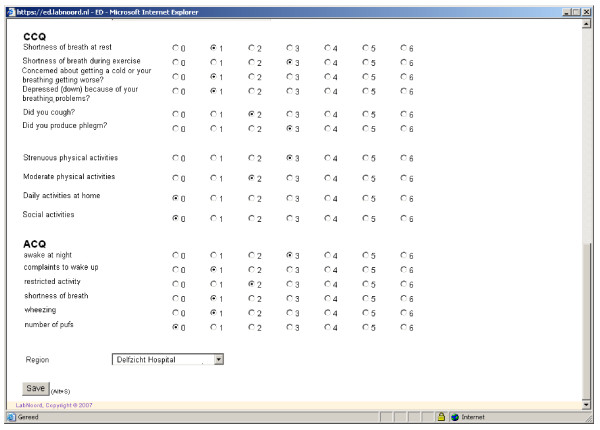
**Assesment of health status by CCQ and ACQ**.

**Figure 2:**
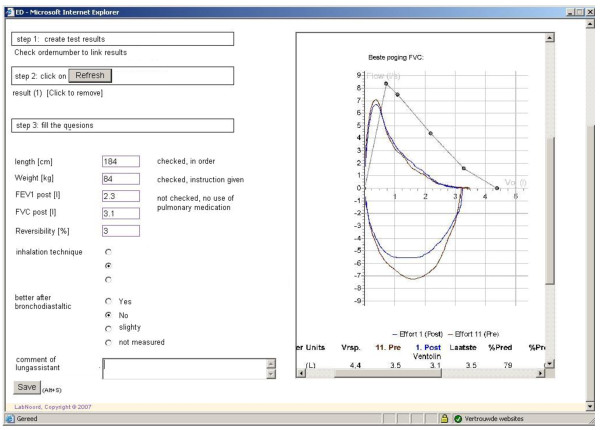
**Spirometric values and inhalation technique**.

**Figure 3:**
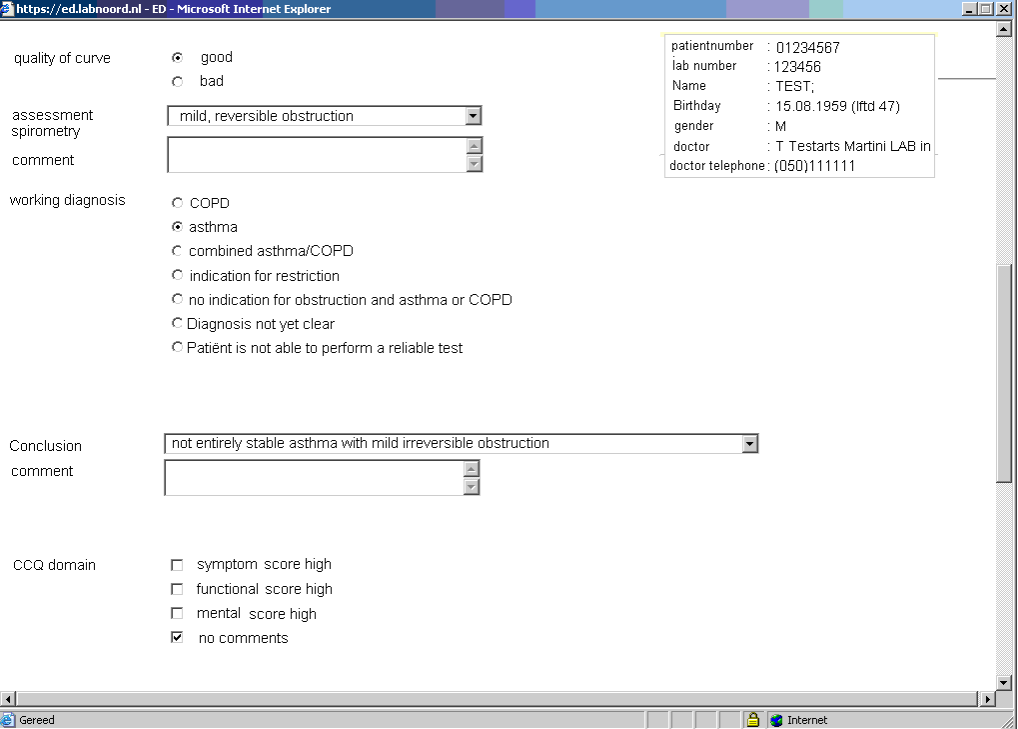
**The service provide GP a diagnosis**.

**Figure 4:**
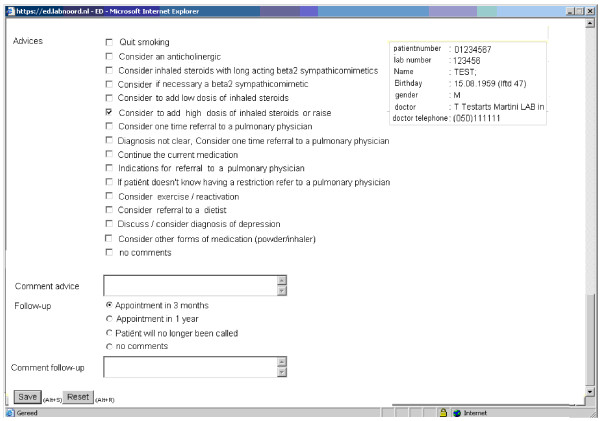
**The pulmonologist gives precise advice to the general practitioner**.

The design of this COPD-asthma service is based on the Dutch COPD-asthma service (prototype) developed by the Northern Laboratory service for GPs (Labnoord) in cooperation with the UMCG (University Medical Centre Groningen) in Groningen, The Netherlands. The service has been adapted to the Greek context. The English version of the main flow-charts governing the software interface (part of the service) can be seen in figures 1,2,3,4.

### Participants

Ten General Practitioners from around Crete and three Pulmonologists from University Hospital in Heraklion, Crete, will be recruited into the study. During the course of the study, we will also recruit up to 20 respiratory patients who have been seen through the service. The patients will be sampled purposively to enable as wide a range of views as possible e.g. asthma/COPD, range of severities, from straightforward cases to those who were referred to secondary care via the service.

This telemedicine project is considered a new technology for Greece so further issues, such as appropriateness, clearness, adherence, compliance, patient and doctor's satisfaction will be estimated with the use of interviews.

### Procedures

The general practitioners and pulmonologists will attend a joint training of two full days on the asthma-COPD telemedicine service.

The steps of the procedure that will be followed regarding the application part are described in Table 1. In addition to spirometry, patients will have to complete three short questionnaires either at home or shortly before the spirometry. The first questionnaire (Table 2) that would be used contains a limited number of items about medication (dosage-frequency), employment, smoking behavior, exacerbations, history, allergy, hyper-reactivation and family history. Secondly patients will complete the Asthma Control Questionnaire (ACQ) and the COPD clinical questionnaire (CCQ) questionnaires [19,20]. Data are then entered into the software programme by the general practitioner (Figure 1) who will then perform spirometry and check inhalation technique (Figure 2). Spirometry data is automatically entered into the computer via the spirometer. All data will then be available real-time to the Consulting Pulmonologist through the network.

**Table 1:** Steps regarding the procedure.

Step 1. First visit questionnaire will be feedeed to the system.
Step 2. CCQ and ACQ scores will be incorporated to the system.

Step 3. Spirometric values and curve will enter the system (example fig 2).

Step 4. Supported by the built of the service based in guidelines, the GP will receive diagnosis and management support.

Step 5. All the previous details will be sent to pulmonologists whenever needed.

Step 6. The pulmonologists will reply with a diagnosis, advice and recommendations for treatment.

Step 6. The general practitioner will have the pulmonologist assessment and clear advices about diagnosis and therapy.

Step 7. Second visit. Assessment of the medicine and patient compliance. The system will give the opportunity for comparison between previous and current results.

Step 8. If needed the GP will ask for new specialist assessment, and then will manage the patient.

**Table 2:** Questionnaire used for the assessment of history, smoking habit, family history and treatment.

1^st ^visit Questionnaire		
**1. What medication do you use?**		
□ None I do not use medication		
□ Yes,		
Name medicine	Dosis	Frequency

		

		

		

**2. Have you ever smoked for longer than 1 year?**		
□ Never		
□ Yes but I stopped smoking since: ..............		
□ Yes I smoke		

**3. How often last year did you receive antibiotics and/or prednisolone prescribed because of your pulmonary problems ? **...................times		

**4. At which age did your pulmonary problems appear for the first time? **I was ..... (age) years old.		

**5. Have you ever been diagnosed with eczema, hay fever, allergy, asthma, COPD or bronchitis?**		
□ No		
□ Yes (please mark the subject)		

**6. Does your family (parents, brothers, sisters) suffer from pulmonary problems?**		
□ No		
□ Yes		
□ I do not know		

**7. What is or was your occupation?** ....................................................................................		

**8. Due to what or when do you experience symptoms such as being short of breath, wheeze, mark if yes**.		
□ springtime	□ wheeds	□ cigarette smoke
□ summer	□ trees	□ paint
□ food	□ cold air	□ perfume
□ pet animals	□ fog	□ exercise
□ dust (house)	□ cooking scent	□ other: :...............

The GP and the pulmonologist will have access to the flow charts on screen containing patients' data, spirometric values and health status assessment. Supported by the built of the service based in guidelines, the GP will receive diagnosis and management support. The pulmonologist will go through the system advice with the GP whenever the GP asks for advice i.e. complicated cases. The first step in the process is assessment of the spirometry results and deciding whether the presented flow volume curve is acceptable. The GP then makes the appropriate diagnosis, in consultation with the pulmonologist in less straightforward cases i.e. asthma, COPD, both or currently unknown (Figure 3). In addition to the diagnosis, the pulmonologists can advise on medication changes, exercise, or assessment of depression. The pulmonologist will give in details, advices about the treatment and will, in conjunction with the GP, determine the time of the next follow-up (Figure 4). If the diagnosis is not certain, the pulmonologist will suggest a referral to secondary care for a more expert approach. The GP will continue the follow-up and can ask for further assistance from the pulmonologist, if needed.

In the follow up second visit a flow chart with details about medicine, compliance, smoking behavior, motivation to quit and health status will be assessed. The system will allow for comparisons in spirometric values, health status, body mass index (BMI) and symptoms from initial visit to follow up visit. Similarly to the first visit, the GP will have the opportunity to ask the pulmonologists for a second assessment and further advice, if necessary.

### Evaluation/analysis

A qualitative approach will be used to evaluate the study, using interviews and focus groups [21]. GPs (n = 10) and patients (n = 20) will each take part in focus groups. Individual interviews will be offered for those who are unable to attend the scheduled focus group (either face to face or by telephone). The pulmonologists (n = 3) will also be interviewed. Topics to be explored will cover; training, use of the technology, use of the flow charts, experience of using the telemedicine service, barriers and problems encountered and acceptability of the process from all view points. The topic guide is currently in development. Both interviews and focus groups will be recorded and transcribed.

The data from the interviews and focus groups will be coded descriptively and analysed using the framework approach to map out the range of experiences and views and to also aid in comparisons between participants (especially those with different experiences of attitudes towards the service) [21,22]. Data from the questionnaires and spirometry will also used to supplement the qualitative data.

## Conclusion

In Greece, a tailored asthma-COPD telemedicine service has the potential to be of great help to GPs and could spare patients potentially long trips across the sea to the secondary care. Furthermore, it may also reduce feelings of professional isolation experienced by GPs and improve access to, and communication with, secondary care colleagues. It is anticipated that it will improve early diagnosis and appropriate management of asthma and COPD in primary care settings in Crete, Greece.

## List of abbreviations

GP: General Practitioners; COPD: Chronic obstructive pulmonary disease; CCQ: COPD clinical questionnaire; ACQ: Asthma control questionnaire.

## Competing interests

The authors declare that they have no competing interests regarding this manuscript. Doctors that will participate in the study are employees of the Greek National Health System and they are paid by a monthly salary, independently of interventions. Indeed there are no financial consequences of reporting positive or negative findings regarding this particular telemedicine program except maybe if the NHS will decide to expand it in case of positive results.

## Authors' contributions

IGT translated the system interface, and prepared the first draft, CL revised the first draft. IGT, TvM, NET, NMS, EvH, and CL have all participated in conception and design, helped in drafting, and gave relevant comments. All authors have given their final approval of this version to be published.
